# Depression, anxiety, and burnout among hospital workers during the COVID-19 pandemic: A cross-sectional study

**DOI:** 10.1371/journal.pone.0276861

**Published:** 2022-12-09

**Authors:** Andrea Norcini Pala, Jessica C. Chuang, Ai Chien, David M. Krauth, Stefano A. Leitner, Nnenna M. Okoye, Sadie C. Costello, Robert M. Rodriguez, Lila A. Sheira, Gina Solomon, Sheri D. Weiser

**Affiliations:** 1 Columbia School of Social Work (CSSW), New York, NY, United States of America; 2 San Francisco (UCSF) Division of Occupational and Environmental Medicine, University of California, San Francisco, CA, United States of America; 3 San Francisco (UCSF) Division of HIV, University of California, Infectious Disease and Global Medicine, San Francisco, CA, United States of America; 4 Division of Environmental Health Sciences, University of California, Berkeley, School of Public Health, CA, United States of America; 5 San Francisco (UCSF) Department of Emergency Medicine, University of California, San Francisco, CA, United States of America; Gachon University Gil Medical Center, REPUBLIC OF KOREA

## Abstract

**Objectives:**

Healthcare personnel have faced unprecedented mental health challenges during the COVID-19 pandemic. The study objective is to assess differences in depression, anxiety, and burnout among healthcare personnel with various occupational roles and whether financial and job strain were associated with these mental health outcomes.

**Methods:**

We employed an anonymous survey between July and August 2020 at an urban county hospital in California, USA. We assessed depression, anxiety, and burnout using validated scales, and asked questions on financial strain and job strain. We performed logistic and linear regression analyses.

**Results:**

Nurses (aOR 1.93, 95% CIs 1.12, 3.46), social workers (aOR 2.61, 95% CIs 1.35, 5.17), service workers (aOR 2.55, 95% CIs 1.20, 5.48), and administrative workers (aOR 2.93, 95% CIs 1.57, 5.61) were more likely than physicians to screen positive for depression. The odds of screening positive for anxiety were significantly lower for ancillary workers (aOR 0.32, 95% CIs 0.13–0.72) compared with physicians. Ancillary (aB = -1.77, 95% CIs -1.88, -0.47) and laboratory and pharmacy workers (aB -0.70, 95% CI -1.34, -0.06) reported lower levels of burnout compared with physicians. Financial strain partially accounted for differences in mental health outcomes across job categories. Lack of time to complete tasks and lack of supervisory support were associated with higher odds of screening positive for depression. Less job autonomy was associated with higher odds of screening positive for anxiety and higher burnout levels.

**Conclusions:**

We found significant disparities in mental health outcomes across occupational roles. Policies to mitigate the adverse impact of COVID-19 on health workers’ mental health should include non-clinical staff and address financial support and job characteristics for all occupational roles.

## Introduction

On January 30, 2020, the World Health Organization (WHO) declared the global coronavirus disease 2019 (COVID-19) outbreak a public health emergency of international concern [1]. Hospitals worldwide faced significant challenges as the number of cases increased, the supply of personal protective equipment became scarce, and intensive care units were over capacity [2]. This placed essential workers in healthcare institutions in stressful working environments [3]. In March 2020, the WHO issued statements warning of the potential rise in psychological stressors among healthcare workers [4]. Assessment of mental health including depression, anxiety, and burnout is critical to inform policies and workplace interventions.

Several studies have documented the negative impact of the pandemic on mental health outcomes among the general population as well as frontline clinical providers [5–10]. Globally, the prevalence of depression, anxiety, and stress in the general population was 29.6%, 31.9%, and 33.7%, respectively during the pandemic, according to a recent meta-analysis [10]. In Wuhan, China, during the first wave of the pandemic, a high proportion of physicians and nurses experienced at least mild symptoms of depression (50.4%), anxiety (44.6%), and distress (71.5%) [11]. In a study among 467 healthcare workers in Quebec, Canada, more than half of the sample (51.8%) experienced burnout at least once a week [12]. Similar rates of burnout (49%) and depression/anxiety (38%) have been found in a large sample of physicians and nurses (n = 20,947) in the US [13]. While the majority of the studies have focused on physicians and nurses, little is known about the impact of the pandemic on mental health outcomes of other healthcare workers including, for example, non-clinical staff (e.g., janitorial and food services staff).

Financial strain resulting from the COVID-19 pandemic may have affected mental health outcomes. In the general population, financial strain is a key risk factor for anxiety and depression [14–16]. During the pandemic, many healthcare workers faced financial strain due to unprecedented job losses and financial pressures on some of the US health sectors and healthcare systems [17]. Between March and April 2020, there were 1.5 million jobs lost in the healthcare industry [18]. Minority, immigrant, and less-educated individuals were disproportionately affected by job loss, food insecurity, and delays in medical care [19]. Financial strain might be one of the mechanisms through which the COVID-19 pandemic affected mental health outcomes of essential workers at healthcare institutions.

Job-related stressors can exacerbate stress associated with the COVID-19 pandemic, increasing the risk for poorer mental health outcomes [20]. Stressors such as lack of participation in decision-making, lack of control over work processes and environment, and lack of supervisory support [21] negatively influence workers’ personal or professional life, thereby affecting mental health outcomes [22]. Job-related stressors affect physicians and nurses as well as other staff members including staff supporting clinical services (e.g., pharmacy, laboratory, and social workers) and non-clinical service workers (e.g., janitorial and food services staff). Yet, the association between job-related stressors and mental health outcomes among the clinical and non-clinical staff, particularly during the COVID-19 pandemic, remains poorly understood. There is also limited research on mental health outcomes among essential workers based at safety net hospitals.

To address gaps in the literature on mental health outcomes in healthcare workers during the pandemic, we conducted a cross-sectional study among a comprehensive sample of healthcare personnel (HCP) at Zuckerberg San Francisco General Hospital (ZSFG), a large academically affiliated hospital in California in July and August 2020. Our goal was to explore 1) mental health outcomes across different occupational roles; 2) whether financial strain explained such differences; and 3) whether job-related stressors (i.e., lack of time to complete work, supervisory support, and job autonomy) were associated with mental health outcomes.

## Methods

### Study design & study sample

We conducted this cross-sectional study at ZSFG, a public hospital in San Francisco, CA. From July 23, 2020 to August 31, 2020, we invited ZSFG HCP to participate in an anonymous survey via an email survey link that was embedded in the required daily online symptom screening questionnaire, daily hospital electronic newsletter, and on flyers posted at the single-entry point to the hospital. Eligibility criteria included being an essential HCP who reported to work in person during the recruitment period. The survey and informed consent were available in English, Spanish, Tagalog, and Chinese to avoid selection bias of only English-speaking participants. We obtained written informed consent from all participants for inclusion in the study. We used Qualtrics provided by the University of California, San Francisco (UCSF) to administer and collect the data. To minimize selection bias in favor of Internet-savvy participants, we conducted specific outreach to provide the option to complete a paper version of the survey where relevant. After completion of the survey, participants had the option to enter a raffle drawing for a $300 gift card.

### Ethics approval

This study was deemed exempt from ethics approval from the University of California San Francisco Institutional Review Board (IRB # 20_3446). Written informed consent was obtained from participants.

### Measures

The survey covered basic sociodemographic variables, professional roles and responsibilities, job characteristics such as the amount of supervisory support provided, experiences with COVID-19 and with personal protective equipment, and mental health outcomes. We pilot tested our preliminary instrument on eight physicians and three nurses to ensure understanding and a completion duration of 10 minutes.

### Mental health outcomes

To minimize participant burden, we used the 2-item versions of the Patient Health Questionaire-2 (PHQ-2) [23] and Generalized Anxiety-2 (GAD-2) [24] to screen for depression and anxiety. The PHQ-2 and GAD-2 items were rated on a 4-point answer scale ranging from *Not at all* (0) to *Nearly every day* (3). Participants were considered to screen positive for depression and/or anxiety if their PHQ-2 and/or GAD-2 scores were ≥ 3. To assess occupational burnout, we used the 9-item Mayo Clinic Physician Well-Being Index (MPWBI) [25–28]. Seven items of the MPWBI were rated on a binary, *No* (0)/*Yes* (1) scale. Two items were rated on a 7-point, Likert-type scale. These items were recoded into 3-point variables ranging from -1 to +1. We calculated Cronbach’s alpha using polychoric correlation matrix (i.e., ordinal alpha), which indicated optimal internal reliability of the scale (Ordinal α = 0.92) [28]. The MPWBI burnout assessment was analyzed as a continuous variable.

### Primary explanatory variable

Job categories included physicians, nursing, ancillary staff (e.g., physical therapists, occupational therapists, dieticians), social workers (e.g., behavioral health clinicians, interpreters), laboratory and pharmacy workers, service workers (e.g., housekeeping, food, and nutrition workers), and administrative staff (e.g., clerks, administrators; See S1 Table). Job categories were analyzed as a categorical variable, with physicians as the reference group.

The Financial Strain index included four items that assessed employees’ concerns about their ability to pay housing costs, normal monthly bills, medical bills for regular healthcare, and fear of losing a job or income. The items included: *How worried are you about (1) not being able to pay your rent*, *mortgage*, *or other housing costs*?*; (2) not being able to pay your normal monthly bills (e*.*g*., *utilities*, *phone*, *student loans*, *car payment*, *etc); (3) not being able to pay medical costs for normal healthcare; and 4) losing your job or income*? The responses were on a 5-point scale from *Not at all worried* (1) to *Very worried* (5). Higher scores reflected greater financial strain. The index internal reliability was optimal (Ordinal α = 0.85).

### Predictor

Job Strain was assessed using the following items: *In the past month*, *I had enough time to get my job done*; *How often were you allowed to make a lot of work-related decisions on your own*, which assesses perceived job autonomy; and *How often were you able to count on your supervisor or manager for support when you need it* to assess lack of supervisory support. The first item was rated on a 5-point scale from *Strongly disagree* (4) to *Strongly agree* (0). The remaining two items were rated on a scale ranging from *Never* (4) to *Always* (0). Higher scores for each of these items corresponded to higher job strain.

### Statistical analysis

We conducted statistical analyses using R Studio version 4.0.3 [29]. Descriptive statistics included mean and standard deviation (SD) for continuous variables (e.g., years employed at ZSFG); median and interquartile range (IQR) for ordinal variables (i.e., age and the job strain variables; count and percentage for nominal variables (e.g., race). We used ANOVA and Kruskal-Wallis to test for differences in financial and job strain across job categories, respectively. Post-hoc pairwise comparisons were performed using Holm’s correction to reduce familywise error rate inflation. We performed multivariable logistic and linear regression analyses to analyze binary (i.e., PHQ-2 and GAD-2) and continuous (i.e., burnout) mental health outcomes, respectively. We used Maximum Likelihood estimation and full information maximum likelihood (FIML) to handle missing data. All regression models were adjusted for covariates determined *a priori* to be associated with the independent and dependent variables, including age, gender, years employed at ZSFG, household size, race, and ethnicity. Statistical significance was set at *p* ≤ 0.05. We evaluated the potential role of financial strain in explaining the differences in mental health outcomes between job categories. First, we ran the regression analysis (logistic and linear) with job category as the main predictor and adjusted for sociodemographic covariates (without financial strain). Then, we included financial strain in the model with job categories and sociodemographic covariates. Changes in the association between job categories and mental health outcomes between the two models were interpreted as a potential mediating role of financial strain.

### Patient and public involvement

Patients and the public were not involved in the design, conduct, reporting, or dissemination plans of our research.

## Results

### Sample characteristics

We reached a sample of 1,171 essential workers who worked on site and answered our survey during the study period, out of a total of an estimated 2,277 employees working on-site during the time of the study. The total number of workers who were on site was not available; we estimated this with the support of the human resources department by excluding individuals who were assumed to be telecommuting based on their roles. The median age of respondents was 40 years (IQR: 33, 50) and most (73.8%) were female. The sample was predominantly Asian (47.4%) or White (38.4%). Nursing comprised the largest group (33.7%). The average duration of employment at ZSFG was 2.55 years (SD = 1.77; see Table 1).

**Table 1:** Participant demographics & baseline characteristics.

	Overall (n = 1,171)
**Demographics**	
Age, median (IQR)	40 (33, 50)
Female, n (%)	841 (73.8)
Race,^*I*^ n (%)	
*White*	361 (38.4)
*Asian*	446 (47.4)
*Black*	69 (7.3)
*Latinx/Hispanic*	180 (15.4)
*Other*	64 (6.8)
Household Size, mean (SD)	3.11 (1.80)
**Job Characteristics**	
Years employed at ZSFG, mean (SD)	2.55 (1.77)
Job Category,^*II*^ n (%)	
*Physician*	162 (14.1)
*Nursing*	388 (33.7)
*Ancillary*	78 (6.8)
*Social Service*	125 (10.9)
*Lab & Pharmacy*	98 (8.5)
*Service Workers*	145 (12.6)
*Administrative*	154 (13.4)
**Mental health**	
Depression (PHQ-2), mean (SD)	0.25 (0.43)
Positive Depression Screening (PHQ-2), n (%)	292 (25.3)
Anxiety (GAD-2), mean (SD)	0.29 (0.45)
Positive Anxiety Screening (GAD-2), n (%)	334 (28.9)
Burnout, mean (SD)	2.53 (2.47)
**Financial strain, mean (SD)**	5.47 (3.49)
**Job strain**	
Lack of Time to Get Work Done, median (IQR)	1.5 (1, 3)
Lack of Job Autonomy, median (IQR)	1 (1, 2)
Lack of Supervisory Support, median (IQR)	1 (0, 2)

*Key*: *SD—standard deviation; PHQ-2 = Patient Health Questionnaire-2; GAD-2 = General Anxiety Disorder scale 2;* ^*I*^ *51 participants did not answer or declined to answer;* ^*II*^*21 participants did not answer; Lack of time to get work done*, *job autonomy*, *and supervisory support ranged from 0 to 4*.

### Financial and job strain by job categories

We performed bivariate analyses to examine the differences in financial strain (ANOVA) and job strain (Kruskal-Wallis test) by job category (Table 2). Overall, physicians reported lower financial strain compared with all other job categories, whereas service workers reported the highest financial strain scores. We found statistically significant differences in job strain characteristics across the job categories. Nurses, ancillary clinical staff, and social service workers reported significantly less time to get work done compared with service workers. Nurses also reported less time to get work done compared with physicians and administrative employees. Lack of job autonomy was significantly higher for service workers compared to the other job categories, while social service employees experienced statistically lower scores in job autonomy compared to physicians. Overall, physicians and administrative staff reported the highest levels of lack of supervisory support.

**Table 2:** Financial and job strain levels by job category–bivariate analysis.

	Physician^a^	Nursing^b^	Ancillary^c^	Social Services^d^	Lab and Pharmacy^e^	Service Workers^f^	Admin^g^	*Test(df)p-value*
**Financial Strain, mean (SD)**	3.60(2.76)^f^	5.41(3.43)^a,f^	5.97(3.45)^a,f^	5.89(3.54)^a,f^	5.13(3.24)^a,f^	7.19(3.64)^a^	6.07(3.17)^a,f^	*F = 16*.*43(6*,*1143) p<0*.*001*
**Lack of Time to Get Work Done, median (IQR)**	1(1, 2)	2(1, 3)^a,f,g^	2(1, 3)^f^	2(1, 3)^f^	1(1, 3)^f^	1(0, 1)	1(1, 2)^f^	*χ*^*2*^ *= 48*.*71(6) p<0*.*001*
**Lack of Job Autonomy, median (IQR)**	1(1, 1)^f^	1(1, 1)^f^	1(1, 2)^f^	1(1, 2)^a,f^	1(1, 2)^f^	2(1, 3)	1(0, 1)^f^	*χ*^*2*^ *= 52*.*61(6) p<0*.*001*
**Lack of Supervisory Support, median (IQR)**	0(0, 1)	1(0, 2)^a,g^	1(0, 2)	1(0, 2)^a,g^	1(0, 2)^a,g^	1(0, 2)^a,g^	0(0, 1)	*χ*^*2*^ *= 63*.*88(6) p<0*.*001*

*Key*: *IQR = interquartile range; SD = standard deviation; F*: *F-statistic*, *ANOVA; χ*^*2*^: *Kruskal-Wallis chi-squared test; The letters in superscript identify statistically significant (p<0*.*05) post-hoc comparisons*, *conducted using Holm’s correction for multiple comparisons; Each job category has been associated with a letter in the first row of the table; Lack of time to get work done*, *job autonomy*, *and supervisory support ranged from 0 to 4*.

### Job category and mental health outcomes

We fit multivariable regression models to test the association between job category and mental health outcomes. These models were adjusted only for sociodemographic covariates (i.e., financial strain was not included in the models). The odds of screening positive for depression were significantly higher for nurses (aOR = 1.93, 95% CIs 1.12, 3.46), social service employees (aOR = 2.61, 95% CIs 1.35, 5.17), service workers (aOR = 2.55, 95% CIs 1.20, 5.48), and administrative staff (aOR = 2.93, 95% CIs 1.57, 5.61) compared with physicians. Ancillary workers were less likely than physicians to screen positive for anxiety (aOR 0.45, 95% CIs 0.19, 0.98). Finally, burnout levels were significantly lower among ancillary workers (aB = -0.77, 95% CIs -1.50, -0.05).

### Job category, financial strain, and mental health outcomes

We included financial strain and sociodemographic covariates in the regression models to test whether financial strain contributed to explaining the differences in mental health outcomes between job categories. The results (with and without financial strain) are reported in Table 3. Higher financial strain levels were significantly associated with higher odds of screening positive for depression (aOR = 1.13, 95% CIs 1.08, 1.19) or anxiety (aOR = 1.16, 95% CIs 1.10, 1.21), and elevated levels of burnout (aB = 0.20, 95% CIs 0.15, 0.25). The inclusion of financial strain changed some of the associations between job categories and mental health outcomes. While the odds of screening positive for depression were no longer statistically significant for nursing and service workers, social service employees (aOR = 2.10, 95% CIs 1.06, 4.21) and administrative staff (aOR = 2.29, 95% CIs 1.21, 4.43) were still more likely than physicians to screen positive for depression. The odds of screening positive for anxiety among ancillary workers compared to physicians decreased from 0.45 to 0.32 (95% CIs 0.13, 0.72) when the model was adjusted for financial strain. Likewise, the differences in burnout levels decreased from -0.77 to -1.17 (95% CIs -1.88, -0.47) for ancillary workers compared to physicians and from aB = -0.52 (95% CIs -1.18, 0.15) to aB = -0.70 (95% CIs -1.34, -0.06) for laboratory and pharmacy workers compared to physicians. These results indicate that ancillary and laboratory and pharmacy workers have significantly lower levels of burnout compared with physicians, after adjusting for financial strain.

**Table 3:** Associations between job category and mental health outcomes, with and without financial strain (FS)–multivariable analyses.

	Depression (PHQ-2) aOR(95% CI)		Anxiety (GAD-2) aOR(95% CI)		Burnout aB(95% CI)	
	Without FS	With FS	Without FS	With FS	Without FS	With FS
Physicians (Ref.)						
Nursing	**1.93(1.12, 3.46)** ^*^	1.54(0.88, 2.80)	1.01(0.63, 1.63)	0.78(0.48, 1.27)	-0.07(-0.57, 0.42)	-0.42(-0.91, 0.06)
Ancillary	0.96 (0.39, 2.22)	0.73(0.29, 1.73)	**0.45(0.19, 0.98)** ^*^	**0.32(0.13, 0.72)** ^**^	**-0.77(-1.50, -0.05)** ^*^	**-1.17(-1.88, -0.47)** ^***^
Social Service	**2.61(1.35, 5.17)** ^**^	**2.10(1.06, 4.21)** ^*^	1.42(0.78, 2.59)	1.08(0.58, 2.01)	0.50(-0.14, 1.15)	0.15(-0.48, 0.78)
Lab & Pharmacy	1.09(0.49, 2.36)	0.95(0.42, 2.08)	1.09(0.56, 2.05)	0.91(0.46, 1.75)	-0.52(-1.18, 0.15)	**-0.70(-1.34, -0.06)** ^*^
Service Workers	**2.55(1.20, 5.48)** ^*^	1.90(0.88, 4.15)	1.97(0.97, 3.98)	1.38(0.66, 2.84)	0.40(-0.34, 1.14)	-0.09(-0.82, 0.63)
Administrative	**2.93(1.57, 5.61)** ^***^	**2.29(1.21, 4.43)** ^**^	1.72(0.98, 3.03)	1.27(0.71, 2.27)	-0.08(-0.68, 0.53)	-0.51(-1.11, 0.08)
Financial strain	**-**	**1.13(1.08, 1.19)** ^***^	**-**	**1.16(1.10, 1.21)** ^***^	**-**	**0.20(0.15, 0.25)** ^***^

Key: Ref.: Reference Group; PHQ-2 = Patient Health Questionnaire-2; GAD-2 = General Anxiety Disorder scale 2; CI = confidence interval; aOR = adjusted odds-ratio; Adjusted for age, gender, race, and years worked. aB = adjusted B coefficient; CI = confidence interval; Adjusted for age, gender, race, and years worked. ***Bold*** indicates statistical significance; * p ≤ 0.*05*, ** p ≤ 0.*01*, *** p ≤ 0.*001*.

### Job strain characteristics and mental health outcomes

Lack of time to get work done, job autonomy, and supervisory support were significantly associated with poorer mental health outcomes (Table 4). Lack of time to get work done (aOR = 1.19, 95% CIs 1.02, 1.38) and lack of supervisory support (aOR = 1.28, 95% CIs 1.07, 1.53) were associated with higher odds of screening positive for depression. Lack of job autonomy was associated with higher odds of screening positive for anxiety (aOR = 1.21, 95% CIs 1.01, 1.45). Finally, lack of time to get work done (aB = 0.18, 95% CIs 0.04, 0.33), lack of job autonomy (aB = 0.20, 95% CIs 0.01, 0.37), and lack of supervisory support (aB = 0.52, 95% CIs 0.35, 0.71) were associated with higher levels of burnout.

**Table 4:** Association between job strain characteristics and depression and anxiety.

	Depression (PHQ-2) aOR (95% CI)	Anxiety (GAD-2) aOR (95% CI)	Burnout aB (95% CI)
Lack of Time to Get Work Done	**1.19 (1.02, 1.38)** ^*^	1.11 (0.96, 1.28)	**0.18 (0.04, 0.33)** ^**^
Lack of Job Autonomy	1.11 (0.92, 1.34)	**1.21 (1.01, 1.45)** ^*^	**0.20 (0.01, 0.37)** ^*^
Lack of Supervisory Support	**1.28 (1.07, 1.53)** ^**^	1.17 (0.97, 1.00)	**0.52 (0.35, 0.71)** ^***^

Key: GAD-2 = General Anxiety Disorder scale 2; PHQ-2 = Patient Health Questionnaire-2; CI = confidence interval; aOR = adjusted odds ratio; Adjusted for age, gender, race, and years worked. Key: aB = adjusted B coefficient; Adjusted for age, gender, race, and years.

**Bold** indicates statistical significance; * p ≤ 0.05, ** p ≤ 0.01, *** p ≤ 0.001.

## Discussion

Consistent with the growing literature on disparities in COVID-19 and mental health outcomes by race, ethnicity and socioeconomic status [30–32], our study points to disparities in mental health outcomes across occupational roles as a new type of disparity worthy of further attention. Ancillary clinical, social service, laboratory, pharmacy, food and janitorial service, and administrative staff contribute significantly to the success of direct patient care. Our findings show an increased risk for clinically significant depressive symptoms among nurses, social service, other service, and administrative workers when compared with physicians. Financial strain plays an important role in worsening mental health among essential workers, and partially explains the differences in mental health outcomes between job categories. Finally, our findings highlight the importance of job-related factors that cut across all occupational categories, such as supervisory support, job autonomy, and time to get work done.

The increased rates of depressive symptoms among non-physician healthcare workers may be explained, at least in part, by financial strain stemming from the COVID-19 pandemic. Physicians faced lower financial strain than all other job categories. The inclusion of financial strain in our models resulted in modestly attenuated estimates for depression and some heightened estimates for anxiety and burnout. The financial burdens of the pandemic were unequally distributed, even within hospitals. Some staff had hours reduced while others were suddenly working overtime, consistent with the findings that mental health outcomes may have been partially mediated by financial strain. We hypothesize that the financial crisis due to the global pandemic generated additional job-related stress, which in turn affected mental health outcomes of healthcare workers [20]. This is consistent with previous literature showing that household financial strain and food insufficiency are associated with increased depressive symptoms among healthcare workers [11,28]. In one previous study of nursing home workers, household food insufficiency, financial strain, and work-family spillover were associated with increased depressive symptoms [33]. However, simply controlling for a potential mediator in a regression model may not be sufficient if there are uncontrolled shared causes of financial strain and mental health outcomes [34]. For example, spousal job loss could lead to both financial strain and mental health stress and, by controlling for financial strain, we may have induced collider-stratification bias [34].

Regarding anxiety and burnout, we found fewer differences and less consistency across job categories even though financial strain was associated with higher rates of anxiety and levels of burnout. It is possible that although clinical workers such as physicians experienced lower job strain, they experienced more anxiety and burnout because they were pushed to work harder in less safe conditions to meet surge demands. Additionally, the prevalence of anxiety and levels of burnout were lower among ancillary workers compared with physicians, which may be related to decreased ancillary services provided in some departments, such as specialty services or outpatient clinics. Likewise, laboratory and pharmacy workers reported lower levels of burnout compared with physicians.

This study further investigated how specific job characteristics affect mental health outcomes. This study showed that not having enough time, job autonomy, and supervisory support were associated with increases in symptoms of depression, anxiety, and burnout across all groups. This is consistent with previous studies showing that increased job strain is associated with poor mental health outcomes. Both cross-sectional and longitudinal studies have demonstrated that job strain is a risk factor for depression [35,36]. These findings may help institutions address and lessen the impact of poor mental health through workplace reorganization, providing more job autonomy, expansion of job duties, discretion in personal time management, flexibility in work schedule, and independence in making decisions. Additional supportive interventions may include increased supervisory guidance and frequent encouragement by supervisors.

During the 2002 SARS outbreak in China, a systematic literature review identified occupational factors that affected the psychological well-being of healthcare personnel [22,37]. Consistent with these findings, our study results confirm that mental health disparities exist across various occupations within the same institution. Previous studies focused primarily on clinical staff, while our study included non-clinical staff such as service workers, social service workers, and administrative staff who reported more depressive symptoms relative to physicians.

### Limitations

Only ZSFG employees working on-site were reached, which limits the generalizability of the study findings. We reached approximately 51% of ZSFG employees who likely worked on-site during the time of the study, but this response rate is an estimate because the actual number of on-site employees was not known. We hypothesize, however, that the reported response rate is an underestimate of the true response rate, as not all essential workers were necessarily on-site during the study period given that many essential functions could be conducted remotely. Respondents were predominantly Asian and White/non-Hispanic, and therefore results are less generalizable to other racial and ethnic groups. This was a cross-sectional study, which limits the ability to assess causality. Additionally, online surveys are subject to self-report bias, entry errors, and non-response bias. The differences found between job categories in terms of mental health outcomes may not be solely caused by the COVID-19 pandemic. The use of 2-item scales may be helpful to screen for depression and anxiety but does not capture the full spectrum of symptoms, and can not be used to diagnose major depressive disorder or generalized anxiety disorder. Additionally, questions assessing financial strain were non-validated. The study was also conducted at an academically affiliated county hospital with characteristics that may not reflect experiences at non-academic and non-governmental institutions.

## Conclusions

Essential workers at healthcare institutions are critical in providing medical treatment in the community at large, especially during the COVID-19 pandemic. Our findings highlight the importance of monitoring physical and mental wellbeing among all essential workers at health care institutions, not just among doctors and nurses who are the most visible front-line workers. Early identification of psychological distress and burnout as well as increasing access to medical care for employees and family members may help lessen the negative psychological impacts on essential healthcare workers.

Recognition and alleviation of job strain through work reorganization and staff training may also help lessen the impact of poor mental health and improve employee well-being. Providing more job autonomy such as the ability to expand job duties and responsibilities, discretion in personal time management, flexibility in work schedule, and independence in making decisions may also reduce symptoms of poor mental health for the employee. Additional supportive interventions may include peer support systems, increased supervisory guidance, and frequent encouragement by managers and supervisors.

## Supporting information

S1 Table List of all job categories included in our analysis. (DOCX)
