# Supplementary material for: Depression, anxiety, and burnout among hospital workers during the COVID-19 pandemic: A cross-sectional study
Source: PLoS One. 2022 Dec 9;17(12):e0276861. doi: 10.1371/journal.pone.0276861 (PMC9733879; doi:10.1371/journal.pone.0276861)
Supplement: S1 Table — (DOCX) [file pone.0276861.s001.docx]

**Appendix**

**Table S1.**

| **Job Category** |  |
| --- | --- |
| **Physician (Reference Group)** | Doctors of medicine (MD) and doctors of osteopathic medicine (DO) including professors, residents, and fellows |
| **Nursing** | Registered nurse (RN), nurse practitioner (NP), certified nurse assistant (CNA), licensed vocational nurse (LVN), certified registered nurse anesthetist (CRNA), patient care assistant (PCA), lactation consultants, and surgical technicians |
| **Ancillary staff** | Physical therapists, occupational therapists, respiratory therapists, dieticians, phlebotomists, radiology technicians, and dialysis technicians |
| **Social workers** | Behavioral health clinicians, interpreters, Chaplains, eligibility workers, sheriffs, and patient navigators |
| **Laboratory and pharmacy** | Pharmacists, sterile processing technicians, and other technicians who do not have patient interactions |
| **Service workers** | Housekeeping, environmental service workers, maintenance workers, laundry staff, food & nutrition services, painters |
| **Administrative staff** | Clerks, administrator, analysts, coordinators, information technologists, researchers, managers, supervisors, and directors |
